# Image-derived input functions for [$^ {18}$F]LW223 and [$^ {18}$F]SynVesT-1 PET in the rodent determined using an autoencoder (IDIF-AE)

**DOI:** 10.1088/1361-6560/ae6eb0

**Published:** 2026-06-08

**Authors:** Jan C Kutos, Arno von Kietzell, Iona McGowan, Daniele Bertoglio, Mark G MacAskill, Adriana AS Tavares, Catriona Wimberley, Paul S Clegg

**Affiliations:** 1 School of Physics and Astronomy, University of Edinburgh, Edinburgh, United Kingdom; 2 Bio-Imaging Laboratory, University of Antwerp, Antwerp, Belgium; 3 μNeuro Research Center of Excellence, University of Antwerp, Antwerp, Belgium; 4 Edinburgh Imaging, University of Edinburgh, Edinburgh, United Kingdom; 5 Institute of Neuroscience and Cardiovascular Research, BHF-University of Edinburgh, Edinburgh, United Kingdom

**Keywords:** arterial input function estimation, deep learning, autoencoder, pre-clinical imaging, dynamic positron emission tomography (PET), small-animal [^18^ F]LW223 PET

## Abstract

*Objective.* Quantitative analysis of dynamic positron emission tomography (PET) scans requires knowledge of the arterial input function (AIF). Existing means of extracting the AIF are invasive and costly (continuous blood sampling), or come with significant errors (image-derived input function, IDIF). We present a novel image-derived AIF method using a machine learning technique that does not require external training data. *Approach.* Voxel-by-voxel time-activity curves are used as individual input samples for training a customised autoencoder (AE) machine learning model. AEs are models that map input samples to themselves, with an intermediate latent layer with few nodes. This drives the training algorithm to find an optimal bottleneck representation of the input. The IDIF is extracted from the weights of the trained model and normalised using a single timed blood sample. *Main results.* The method was evaluated on dynamic PET scans of rats with translocator protein tracer [$^ {18}$F]LW223. Volumes of distribution ($V_\mathrm{T}$) from arterial blood sampling (ground truth) were compared using Logan plots with IDIF-AE (mean absolute percentage error $\pm32\%$) and conventional IDIF from left ventricle ($\pm54\%$). The method was also successfully adapted for scans of mice with neuro PET tracer [$^ {18}$F]SynVesT-1. *Significance.* This study demonstrates a novel machine-learning based IDIF for dynamic PET that can outperform classical IDIFs in determining kinetic parameter $V_\mathrm{T}$, without requiring external training data.

## 1. Introduction

### 1.1. Image-derived input functions (IDIFs)

Positron emission tomography (PET) is a widely used functional imaging technique in nuclear medicine (Torigian *et al* 2007, Cherry *et al* 2012). PET is most commonly used in diagnostics, for example in the staging and mapping of cancer, but also has significant applications in clinical and pre-clinical research (Cherry *et al* 2012). It is possible to image dynamic processes by acquiring dynamic PET scans (Lammertsma 2017, Dimitrakopoulou-Strauss *et al* 2021), where data is binned into a number of time-frames.

Quantification of dynamic PET kinetics requires knowledge of the (metabolite-corrected) arterial input function (AIF) (Innis *et al* 2007, Zanotti-Fregonara *et al* 2011, Lammertsma 2017). The gold-standard for AIF determination is blood sampling by arterial cannulation (Volpi *et al* 2023). This provides precise activity concentration values, as both activity and extracted blood volume are known. Where required, extracted blood may be centrifuged to perform metabolite correction. While generally a safe procedure, this technique adds significantly to the complexity of required equipment and to patient discomfort, and may discourage some patients from participation in PET studies (Volpi *et al* 2023). In pre-clinical research, rodents are often used as model systems to investigate pathology in PET research. Due to their more limited blood volume, continuous arterial blood sampling often comes with physiological changes (Laforest *et al* 2005). Such experiments are also generally terminal, making longitudinal studies impossible.

To avoid sampling every subject, a population mean input function, optionally adjusted for patient characteristics such as weight and age, can be used (Takikawa *et al* 1993, Zanotti-Fregonara *et al* 2011). However, this approach can mask important differences in individual subject input functions and breaks down when there are systematic differences present, particularly when diseases or therapeutic agents cause meaningful changes in metabolic processes.

To steer clear of the complexities and drawbacks of arterial sampling entirely, a number of techniques have been developed to extract the AIF directly from dynamic PET images (Volpi *et al* 2023), known as IDIFs. Conventional IDIFs are taken from the total blood activity curve in a suitable region, such as the ascending or descending aorta, the left ventricle or, in brain studies, the carotid arteries (Weerdt *et al* 2001, Zanotti-Fregonara *et al* 2009). With few exceptions (such as human total-body PET scanners), a single arterial blood sample must generally still be taken during the scan for calibration (Zanotti-Fregonara *et al* 2009). Recent advances in scanner resolution have also opened the possibility of IDIFs in pre-clinical rodent studies (Lanz *et al* 2014).

However, conventional IDIFs have a large error range due to factors including partial volume effects, patient movement during the scan, and the need to manually delineate the region of interest (Laforest *et al* 2005, Soret *et al* 2007, Volpi *et al* 2023). Where metabolite correction is needed, a population-mean parent fraction is generally used, as metabolite ratios cannot be gleaned from the image alone. The plasma-to-whole-blood ratio can similarly be approximated by a population-mean, but this again adds to the cumulative uncertainty.

As a result, a number of novel approaches have been proposed in recent literature. The conventional IDIF can be fitted to a known parametrised input function model (Kuttner *et al* 2020), but this can erase important peculiarities in individual scans. Statistical analysis techniques such as factor analysis can separate the primary signals in the image without the need for (manual) image segmentation (Laforest *et al* 2005, Kim *et al* 2013), but it is difficult to ascertain that one of these signals is the input function without any mix-in from tissue curves.

Most recently, promising new machine learning-based techniques have been developed. These use a wide range of architectures, including Gaussian processes (Kuttner *et al* 2020, 2021), long short-term memory networks (Kuttner *et al* 2020, Wang *et al* 2024), convolutional neural networks (Wang *et al* 2020, Jiang *et al* 2023, Whitehead *et al* 2023, Kuttner *et al* 2024), physics informed neural networks (Ferrante *et al* 2024), hierarchical clustering (Moradi *et al* 2024) and vision transformers (Chen *et al* 2025). The models are all trained on a set of scans with a ground truth reference AIF (usually from blood sampling) and are then used to predict an input function by invocation on an unseen scan and, in some architectures (Wang *et al* 2020, Jiang *et al* 2023, Whitehead *et al* 2023), patient characteristics and dose information.

These methods exhibit strong performance when used on data which is similar to the training data used. However, when evaluated on scans acquired using different protocols, tracers or scanners, performance drops significantly, implying a need to re-train these models for each novel dataset. A notable exception is the architecture proposed by Vashistha *et al* (2025), which uses variational inference with data from other parts of the same scan rather than external training data. However, this approach relies on a statistical atlas of cerebral arteries and thus is only relevant in humans.

### 1.2. Autoencoders (AEs)

In this paper, we propose a novel IDIF extraction method using a class of neural networks known as AEs. Uniquely, our approach relies only on population-level assumptions about tracer kinetics and does not require any external training data. This makes it particularly suitable for pre-clinical settings, where available data is sparse, pathologies can substantially affect dynamics, protocols change frequently and kinetics may differ significantly from those seen in previous literature.

AEs are a class of neural networks that are trained to reproduce their input as their output, via a set of hidden layers. They have been shown to be particular adept at feature extraction tasks (Bourlard and Kabil 2022). AEs are made up of two parts: an encoder and a decoder. The encoder takes an input feature vector and transforms it to a latent (hidden) representation. The decoder takes the latent representation and transforms it back into the dimensions of the input vector. Models with a latent bottleneck layer of smaller size than the input/output (undercomplete AEs) are generally most adept at salient feature extraction (Bourlard and Kabil 2022).

### 1.3. Datasets

This study used small animal (rodent) scans with a novel translocator protein (TSPO) tracer, [$^ {18}$F]LW223 (MacAskill *et al* 2021a, 2021b). Small animal scans have unique challenges for conventional IDIFs due to the limited spatial resolution (Lanz *et al* 2014). TSPO is an inflammation biomarker and a widely studied PET target. However, clinical adoption of TSPO PET has been hindered by suboptimal properties of existing tracers (Chauveau *et al* 2008), most notably the rs6971 genetic polymorphism in humans, which affects the binding affinity of second-generation TSPO tracers (Owen *et al* 2012). [$^ {18}$F]LW223 is not susceptible to this polymorphism. The tracer is also slowly metabolised in the rodent (MacAskill *et al* 2021b).

Performance was also tested on scans using a novel neuro PET tracer, [$^ {18}$F]SynVesT-1. The concentration of the A isoform of synaptic vesicle glycoprotein 2 (SV2) reflects the density of synapses in different regions of the brain (Bertoglio *et al* 2022). Changes to synapses can be indicative of neurological disorders and ageing. Recently it has become possible to measure the concentration of SV2(A) using PET and the [$^ {18}$F] based tracer [$^ {18}$F]SynVesT-1 (Bertoglio *et al* 2022, Miranda *et al* 2023). Unfortunately [$^ {18}$F]SynVesT-1 is rapidly metabolised in the body. While kinetic parameters can be estimated without measuring the proportion of the unmetabolised tracer that is available in the blood (the plasma parent fraction, PPF), doing so reduces quantitative accuracy (Xiong *et al* 2023).

### 1.4. Aims of the study

In this study, we developed a novel image-based AIF extraction method using an AE, which we termed IDIF-AE. We then evaluated this method on a dataset of fifteen adult rats scanned using [$^ {18}$F]LW223 for an earlier, unrelated study (MacAskill *et al* 2021b) and compared performance to ‘gold-standard’ arterial blood sampling. We also tested performance on a previously acquired dataset of five adult mice scanned using [$^ {18}$F]SynVesT-1 (Bertoglio *et al* 2022).

## 2. Methods

### 2.1. Data

#### [$^ {18}$F]LW223 rats

2.1.1.

The procedure was developed and tested using a dataset of fifteen adult male Sprague-Dawley rats scanned for two hours with novel TSPO tracer [$^ {18}$F]LW223 (MacAskill *et al* 2021a, 2021b). This data was acquired for a previous study. Events were binned into time frames of varying length ($18\times10$ s, $2\times30$ s, $1\times60$ s, $2\times2$ min, $10\times5$ min, $6\times10$ min) and images were reconstructed using 0.4 mm voxels. In nine of the fifteen animals, myocardial infarction (MI) was induced by a previously described method (Spath *et al* 2018); imaging occurred $7\pm1$ days later. The remaining six naive animals underwent no surgical procedures. The rats had food and water available *ad libitum* and a standard 12 h light / 12 h dark regime was employed. Upper-body PET-CT scans were acquired on the Mediso nanoPET/CT scanner after a bolus injection of the [$^ {18}$F]LW223 tracer ($23.1\pm1.7$ MBq). AIFs were determined by a Swisstrace automatic blood sampler.

Experiments were authorised by the local University of Edinburgh Animal Welfare and Ethical Review Committee and in accordance with the Home Office Animals (Scientific Procedures) Act 1986. The animal experiments were conducted and reported in-line with the ARRIVE guidelines.

#### [$^ {18}$F]SynVesT-1 mice

2.1.2.

A separate investigation for the tracer [$^ {18}$F]SynVesT-1 was also carried out. An optimised IDIF-AE protocol for [$^ {18}$F]SynVesT-1 was developed using this data. For these studies, previously acquired total-body scans of five male mice (C5B7BL/6 J) aged 3-4 months were used (Bertoglio *et al* 2022, Miranda *et al* 2023). The mice had food and water available *ad libitum* and a standard 12-h light / 12-h dark regime was employed. $10.0\pm3.5$ MBq ($1.14\pm0.39 \mu$g kg$^ {-1}$) of the [$^ {18}$F]SynVesT-1 tracer was bolus injected via the tail vein at the beginning of the scan. Data were binned into frames of increasing length ($12\times 10$ s, $3\times 20$ s, $3\times 30$ s, $3\times 60$ s, $3\times 150$ s, $21\times 300$ s, total duration 2 h). Images were reconstructed with $0.776\times0.776\times0.796$ mm frames. AIFs were measured using an automatic blood sampler (Swisstrace) and denoised by fitting of a three-exponential function to the decaying part of the curve.

All experiments were performed according to the European Committee Guidelines (decree 2010/63/CEE) and reported in compliance with the ARRIVE guidelines. Experiments were approved by the Ethical Committee for Animal Testing (ECD 2020-59) at the University of Antwerp (Belgium).

### 2.2. IDIF-AE procedure

In the IDIF-AE procedure, a dynamic PET scan is first pre-processed to reduce noise. The voxel-by-voxel time activity curves are then used as individual input samples for training a customised AE machine learning model. Finally, the input function is extracted from the weights of the model and normalised using a single blood sample.

#### 2.2.1. Pre-processing

Two pre-processing steps are applied to the input data. First, the images are manually cropped to remove parts of the body where the input function signal is less present. Two cropping strategies were evaluated: including the head, heart and lungs (HHLs), or including the heart and lungs (HLs) only.

A three-dimensional Gaussian filter is then applied to each frame of the scan separately to reduce random noise. Filters with standard deviations $\sigma$ = 2, 5 and 10 voxels (0.8 mm, 2.0 mm, 4.0 mm) were compared, as well as the absence of a filter.

#### 2.2.2. Model

**Figure 1.**
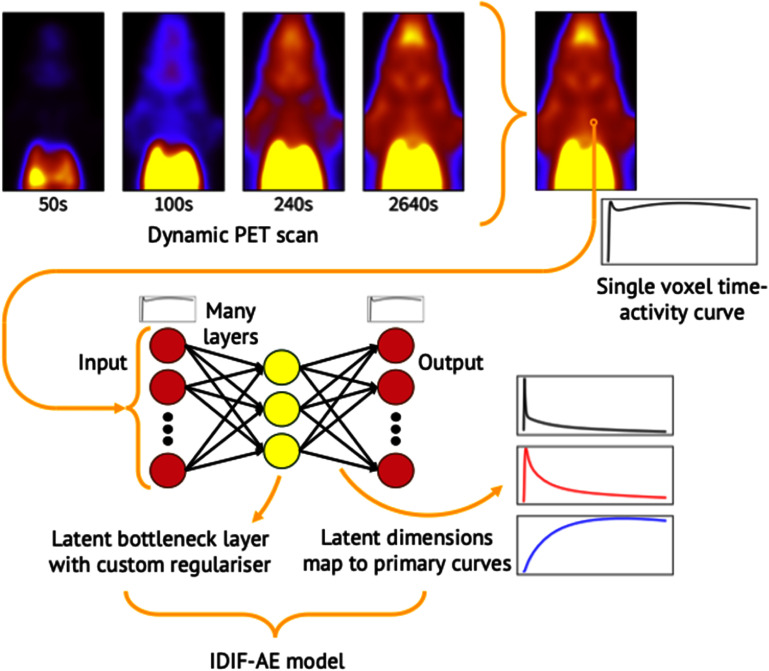
Schematic of the IDIF-AE procedure.

The individual voxel-wise time-activity curves (TACs) from the image are then passed as input samples to the training algorithm of the IDIF-AE model. A schematic of this model is shown in figure 1. The model was implemented in Keras/Tensorflow.

**Figure 2.**
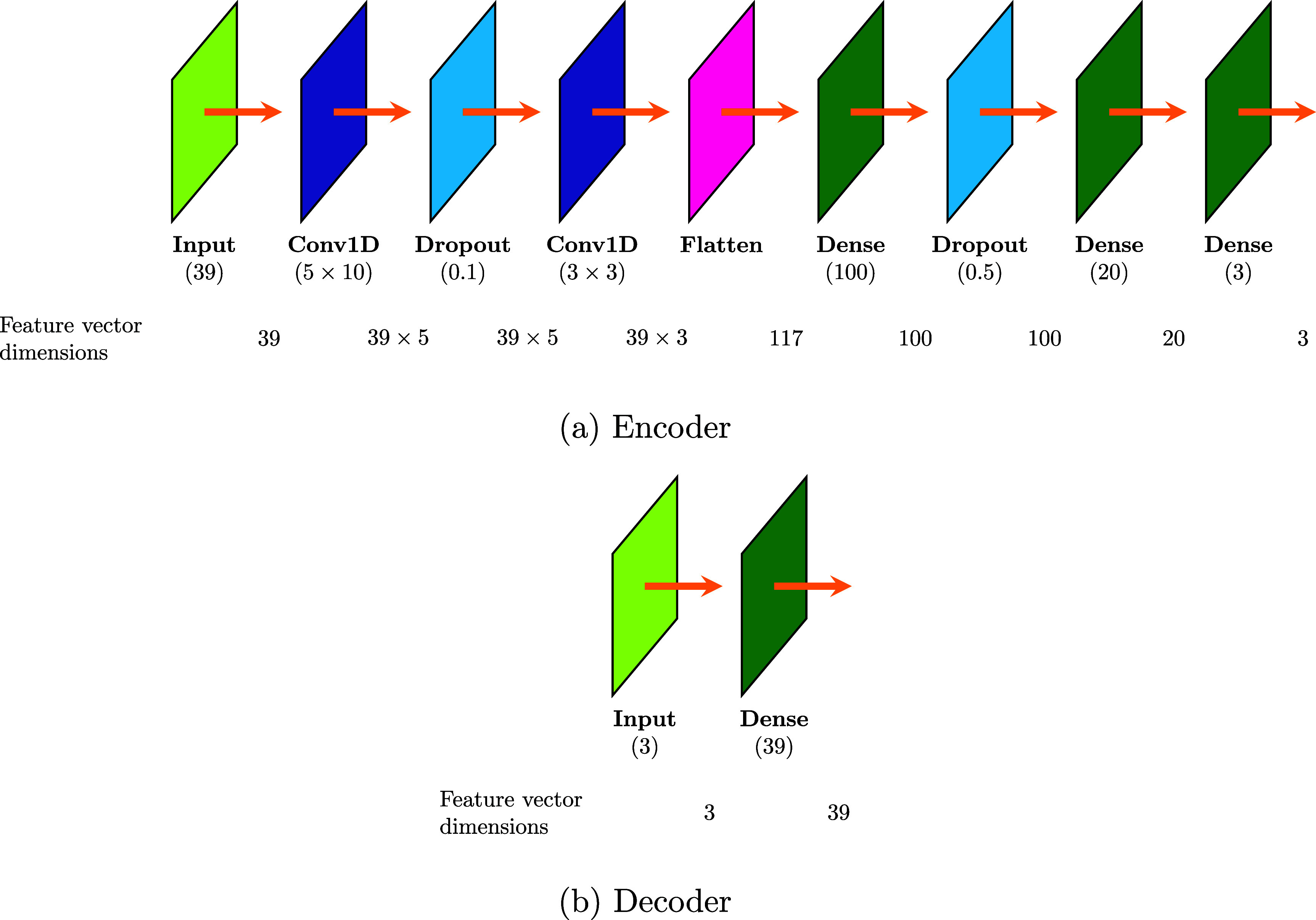
Layers of the IDIF-AE autoencoder model.

The machine learning model underpinning the IDIF-AE is a customised undercomplete AE; the layer structure is shown in figure 2. The encoder consists of a succession of one-dimensional convolutional filters acting in the time dimension, and fully-connected layers. The bottleneck latent space contains three nodes. Finally, the decoder consists of a single fully-connected layer. There is thus a linear mapping from latent to output space.

After the model has been trained, this linear mapping encodes the primary TACs present in the image. In early testing, we found that allowing three primary curves achieved optimal signal separation. As we are working with bolus-injected data, the curve with the earliest peak is assumed to be the input function. Since the curves are averaged across the image, there is no direct physical interpretation for the other two curves.

The signal in one voxel is decomposed as a linear combination

\begin{equation*} S_\mathrm{voxel}\left(t\right) = \sum_{i = 1}^3 w_i \, \phi_i\left(t\right) + \varepsilon\end{equation*}

where $\phi_i$ are the primary curves in the image. The coefficients $w_i$ are the representation of the voxel in latent space; we term them curve contribution factors. $\varepsilon$ represents an error term; model training seeks to minimise the sum of these errors across the image.

In the IDIF-AE method, the AE model is trained in two phases. After the first half of training, the model weights are analysed and the purported input function curve selected. A custom regulariser is then inserted. This modifies the cost function based on the shape of the input function during each training step. The regulariser function must be adjusted for the particular kinetics of each radiotracer. In the current implementation for [$^ {18}$F]LW223, the regulariser penalises curves that do not follow a simple single-peak shape within the first 10 timepoints (75 s); for [$^ {18}$F]SynVesT-1, the weighting of the requirement for the signal to decrease after the peak is weakened. More complex target shapes can readily be implemented. The weighting of the regulariser penality compared to the root-mean-square loss between input and predicted output samples in the AE can also be adjusted, tuning the model between solutions that are closer to the principal components of the image or to physics-based solutions.

Equation (1) is identical to the operational equation of factor analysis (Sitek *et al* 2002). The key advantage of the IDIF-AE method is the addition of the regulariser, which enables us to gradually guide the model towards physically-plausible solutions with each training step.

#### 2.2.3. Post-processing

The IDIF-AE curves are produced dimensionless and cannot easily be normalised using data from the scan (as with conventional IDIFs). Based on initial investigations, normalisation by use of a single arterial blood sample taken one hour after tracer injection was selected to be used for all tracers.

We found that on some occasions, the model could not adequately separate late-scan blood and tissue signals and thus returned physiologically implausible values. This would generally resolve on repeated runs initialised with different random seeds. For this reason, we implemented plausibility constraints based on the ratio between the predicted curve at one hour and the predicted curve peak height (minimum peak:tail ratio 5:1, maximum 15:1). If a curve was returned that did not match these criteria, the run would be repeated with a different seed until the criteria were met.

For determining $V_\mathrm{T}$ for [$^ {18}$F]LW223 data, a population-based metabolite correction was applied to allow for more accurate modelling (MacAskill *et al* 2021a). [$^ {18}$F]SynVesT-1 data was left uncorrected to study the very common case where suitable metabolite curves are not available.

### 2.3. Baseline methods

Conventional IDIFs were obtained using a simplified method for [$^ {18}$F]LW223 (MacAskill *et al* 2021b). A small volume of interest (7 voxels of $0.4\times0.4\times0.4$ mm) in the left ventricle was manually segmented in the centre of the chamber to reduce spill-over effects, but no other partial volume correction was applied.

Factor analysis was carried out on the same set of [$^ {18}$F]LW223 images to provide a performance baseline. This was done using the PIXIES software (Apteryx S.A.R.L., France, www.apteryx.fr) as per Wimberley *et al* (2020), however only a positivity constraint was applied. The variability in the injection protocol (manual injection with a variable peak width) meant that adding a similarity constraint did not yield consistent results and often failed to converge to a solution.

### 2.4. Analysis

#### [$^ {18}$F]LW223

2.4.1.

Initially, we adjusted the parameters of the model to optimise performance for the [$^ {18}$F]LW223 dataset. Important tunable parameters (hyperparameters) in this model include the weighting and temporal region of interest of the custom regulariser, as well as pre-processing steps such as cropping and blurring to reduce noise. The model was optimised to minimise the mean squared difference between IDIF-AE blood curves and corresponding ground truth curves. Performance differences between IDIF-AE and conventional region-of-interest IDIF (MacAskill *et al* 2021b) were visualised at this stage by comparison of the area-under-the-curve (AUC) around the early peak (a window of 7 timepoints centred on the peak).

The extent of overtraining, where the model learns patterns in the image noise rather than the underlying data, was also quantified. For this, a randomly selected subset of 20% of voxels in the scan was put aside and not used in training. The AE model was then invoked on the training data as well as this validation set. Mean root-mean-square errors (RMSEs) between the input voxel curves and the AE output curves were compared. In the general IDIF-AE procedure, all voxels are used for model training.

Machine learning models are initialised with randomised weights, and as such subsequent runs of the method may produce different results. The impact of this was analysed by comparison of the timing of the input function early peak in ten runs with different random seeds; to avoid bias these seeds were chosen to be numbers $1-10$ for all images.

The optimised IDIF-AE model was then used to extract input functions from all 15 scans in the dataset. Logan plotting (Logan *et al* 1990) was used to determine volumes of distribution ($V_\mathrm{T}$) for manually delineated regions of interest (heart and brain, from MacAskill *et al* (2021b)). These were then compared to $V_\mathrm{T}$ values found using the ground truth AIF and the conventional IDIF. Logan plotting $V_\mathrm{T}$ values were also determined using input functions found by factor analysis to act as performance baseline for statistical analysis methods, which are closely related to the IDIF-AE approach.

Finally, the ability of this method to extract usable input functions from brain-only scans was examined. The whole PET scans were divided into 20 axial sections. IDIF-AEs were then extracted and $V_\mathrm{T}$ values found by Logan plotting for the section furthest from the heart, then that and the adjacent section, adding more sections until all 20 were used.

#### [$^ {18}$F]SynVesT-1

2.4.2.

Dynamic mouse PET scans ($n = 5$) using [$^ {18}$F]SynVesT-1 were provided by the University of Antwerp alongside a simultaneously acquired AIF (details in Bertoglio *et al* (2022)) and an IDIF-AE was extracted from the image. The model regulariser was slightly adapted to account for the fact that the blood curve is expected to be higher later in the scan due to formation of radiometabolites. The IDIF-AE was used in an Ito plot (Ito *et al* 2010) to extract $K_1$ and $V_\mathrm{T}$ values for six brain regions (cerebellum, cortex, hippocampus, pons, striatum and thalamus). One animal was excluded due to failed fitting procedures. The radiometabolite-corrected (population based metabolite curve) AIF was also used in a two tissue compartment model (2TCM) as gold-standard comparison values. The values were compared by correlation analysis.

## 3. Results

### [$^ {18}$F]LW223

3.1.

**Figure 3.**
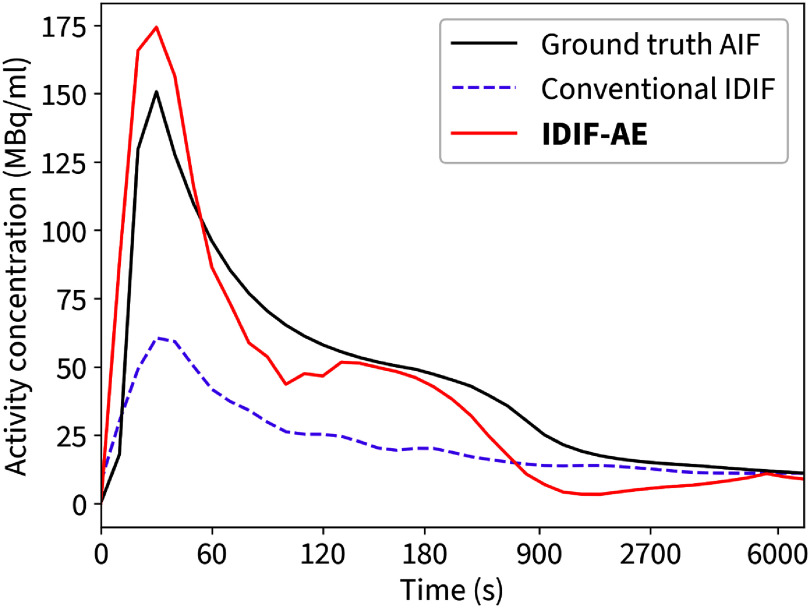
Representative example from one scan: ground truth AIF from arterial blood sampling, naive IDIF found by manual delineation of left ventricle, and IDIF-AE.

Figure 3 shows an example of the IDIF-AE output for a scan in the dataset. The ground truth AIF found by arterial blood sampling and a manually delineated classical IDIF are shown for comparison. The IDIF-AE visually captures the early peak better than the classical IDIF, which was seen frequently across the dataset.

**Figure 4.**
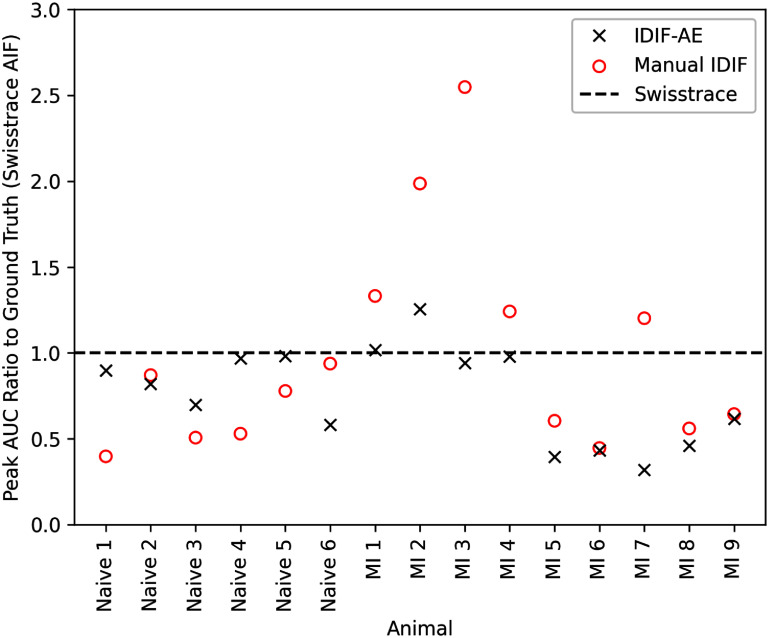
AUC using a window of three timepoints each side around the curve peak, relative to ground truth AIF, for all images in the dataset.

This is illustrated in figure 4, which compares the ability of the conventional IDIF and the IDIF-AE to capture the AUC in a narrow region around the peak across all images in the dataset. The IDIF-AE peak shows substantially improved agreement with the ground truth (mean absolute percentage error 27.9%) compared to the classical IDIF peak (46.9%). For both methods, performance was better on animals that had not undergone MI (IDIF-AE 17.5%, conventional 30.2% vs 34.8%, 56.2% for naive, MI respectively). For reproducibility, however, all values reported here were obtained with pre-selected fixed seeds. In two animals, the initial run produced physiologically implausible results (per section 2.2.3) and a total of 4 re-runs were needed for each animal before a solution meeting the peak-to-tail ratio criteria was found.

**Figure 5.**
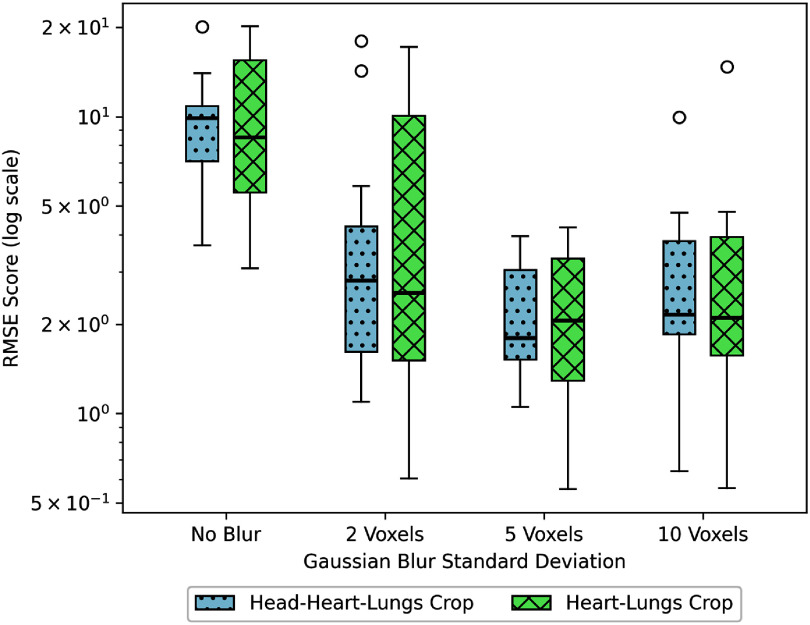
Comparison of RMSE between IDIF-AE curve and ground truth AIF, by image, with different preprocessing. Preprocessing steps are Gaussian blurring and cropping using one of two strategies (HL or HHL).

The results of optimisations of the two preprocessing steps (see methods) are shown in figure 5. Combinations between 4 levels of Gaussian blur (standard deviation $\sigma = 0, 2, 5, 10$ voxels) and two cropping strategies—HHLs or HLs only—were tested for all images and evaluated by comparing the RMSE between the IDIF-AE and ground truth AIF curves. Performance was best for both cropping strategies with blur $\sigma = 5$ voxels (2.0 mm), and therefore further results presented used this blur level. The difference between cropping strategies was small, with mean RMSE scores at $\sigma = 5$ of 87.4 and 89.5 for HHL and HL respectively; thus the head can safely be excluded from the data.

**Figure 6.**
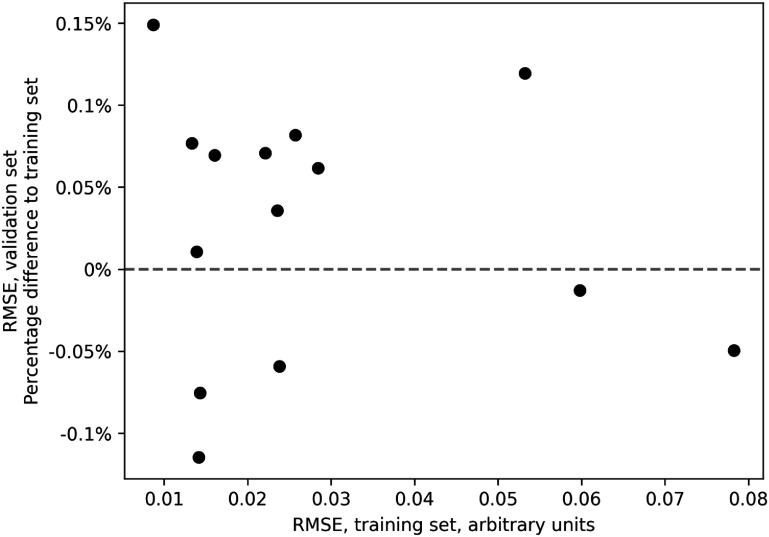
Comparison of RMSE between input and reconstructed output of the autoencoder for training and validation datasets, for each of the 15 scans in the [$^ {18}$F]LW223 dataset.

Figure 6 shows the results of an overfitting analysis on the AE model. It compares the percentage difference in mean RMSE between input and reconstructed output curves, for a training set of 80% of voxels and a randomly selected validation set of 20%, for each image. In all images, this difference was less than $\pm0.15\%$.

**Figure 7.**
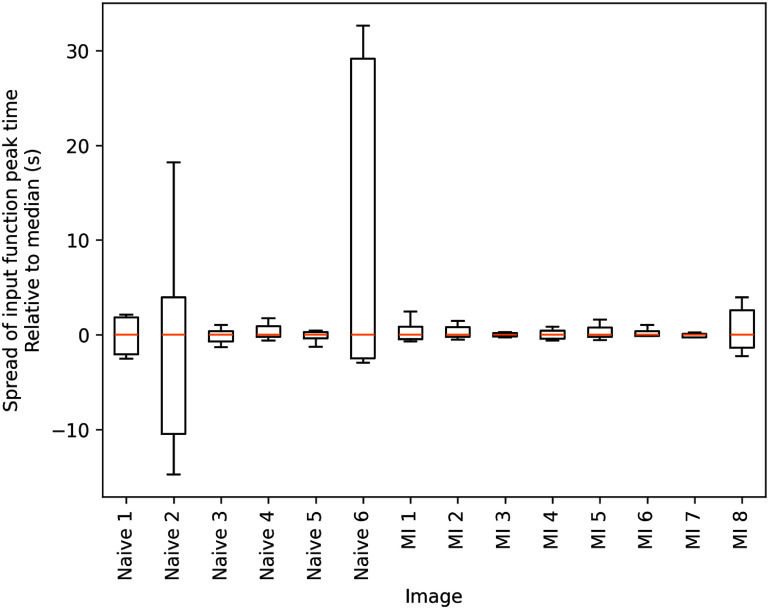
Spread of the peak of the IDIF-AE curve with 10 repeated runs of the procedure, in seconds for scans of total duration 2 h (7200 s).

A reproducibility evaluation is shown in figure 7. This compares the timing of the input function peak for 10 repeated IDIF-AE runs on the same scan with the peak of the mean of all 10 runs. The spread in peak times for all scans was within $\pm35$ s of the mean. For 7 of 15 of the scans, all repeated IDIF-AEs peaked within $\pm1$ s of each other. All scans had a total duration of 7200 s.

**Figure 8.**
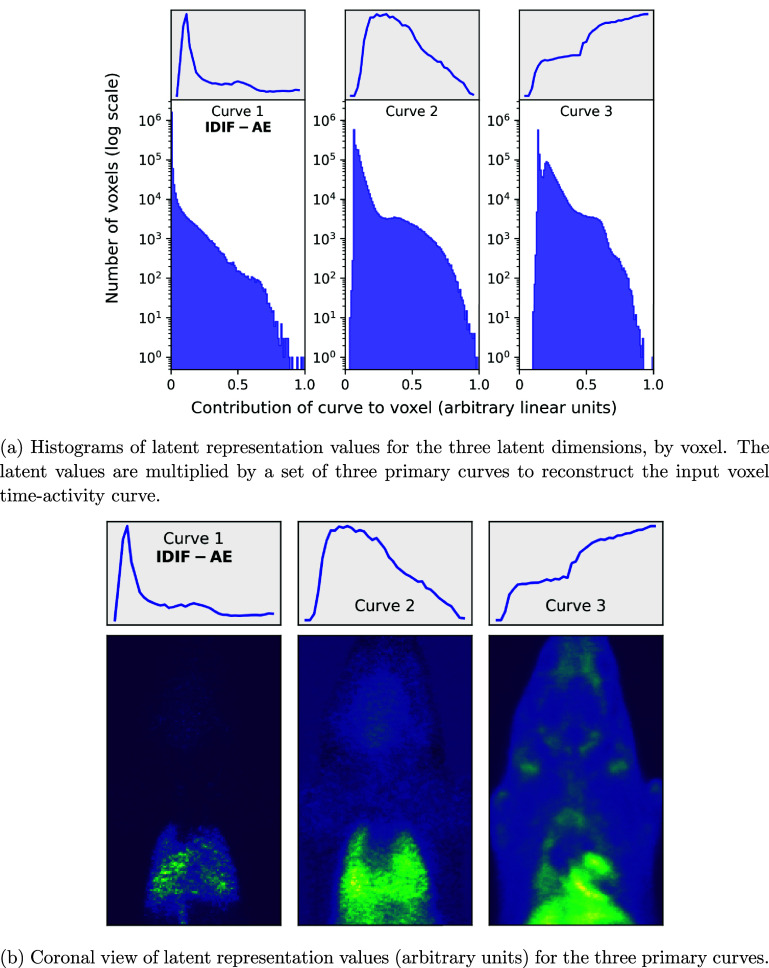
IDIF-AE latent representation values for a representative animal from the [$^ {18}$F]LW223 dataset.

The distribution of latent space weights (contribution factors) across the image was also examined. An example of this is shown in figure 8(a). Contribution factors were scaled such that the greatest value for each factor was 1.0. The logarithmic histograms show the voxel counts associated with varying contributions from each bottleneck representation curve. In this example, the IDIF-AE curve is only present in some voxels, while the other two curves contribute at some non-zero level to all voxels. This was noted in all scans. The histograms were almost unchanged between the first and second half of training.

Figure 8(b) shows the same data on axial slices of the scan. Again, it can be seen that the IDIF-AE curve contributes only to some parts of the image; specifically, contribution is concentrated in the HLs. The other primary curve contributions are spread throughout the image, though signals are still strongest in the HL region.

**Figure 9.**
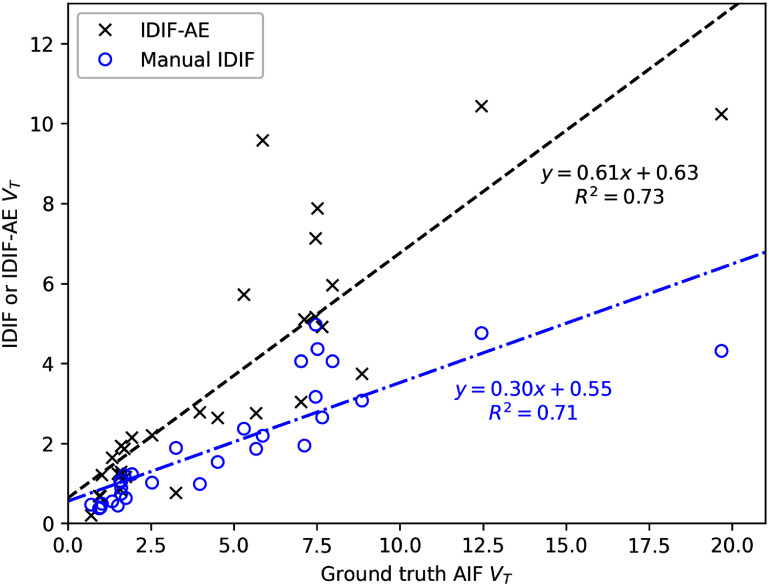
Comparison of brain $V_\mathrm{T}$ values estimated by Logan plotting using the ground truth AIF ($x$-axis) or conventional IDIF/IDIF-AE ($y$-axis), for all 15 images in the dataset. The best-fit line for the IDIF-AE values is $y = 0.61x+0.63$ with coefficient of determination $R^2 = 0.73$. For the conventional IDIF values, this was $y = 0.30x+0.55$ with $R^2 = 0.71$.

For all 15 scans, volumes of distribution $V_\mathrm{T}$ were determined by Logan plotting using the ground truth AIF, IDIF-AE and classical IDIF. A comparison of $V_\mathrm{T}$ values found using the ground truth and IDIF-AE is shown in figure 9, with good linear agreement between the two. IDIF-AE $V_\mathrm{T}$ values were noted to be more prone to underestimate rather than overestimate the true $V_\mathrm{T}$. The mean absolute percentage error in the IDIF-AE $V_\mathrm{T}$ compared to the ground truth across the dataset was $\pm31.4\%$. For the classical IDIF, the mean absolute percentage error was $\pm53.9\%$.

**Figure 10.**
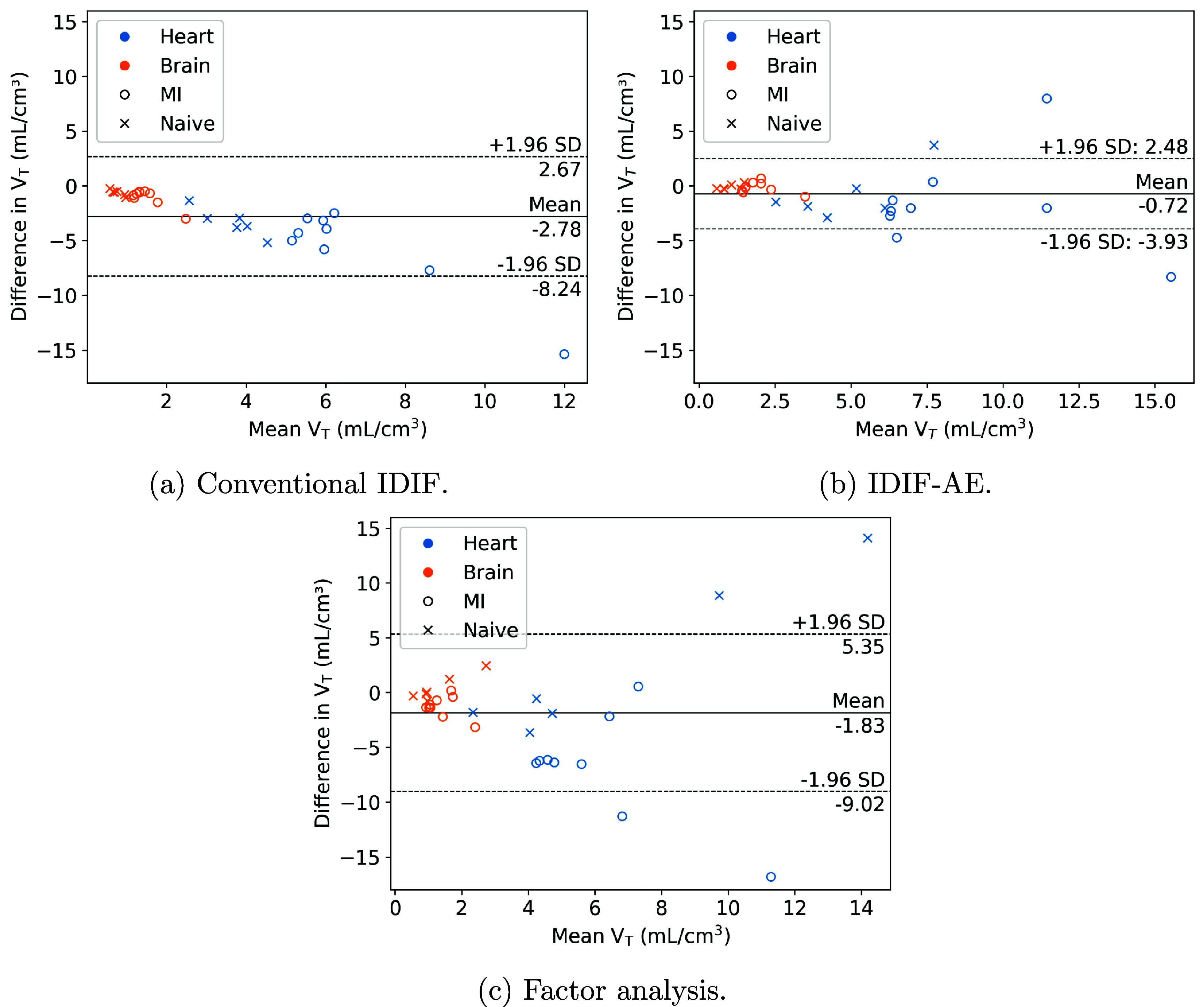
Bland–Altman plots comparing $V_\mathrm{T}$ values derived by Logan plotting using the arterial blood sampling AIF and image-based methods.

Figure 10 shows Bland–Altman plots comparing image-based methods (conventional IDIF, IDIF-AE and factor analysis) with the baseline arterial blood sampling AIF. In all cases, $V_\mathrm{T}$ values were determined using Logan plotting with, for each animal, mean tissue curves for the brain and for the whole heart. IDIF-AE values showed lower bias than the other two methods, and performance was worst for the heart in animals that had undergone MI. Specifically, IDIF-AE mean bias was $-$0.72 ml cm$^ {-3}$ (95% CI: $-$1.54 to 0.10) with limits of agreement from $-$3.93 to 2.48 ml cm$^ {-3}$ (95% CI: $-$4.83 to 3.39). Mean bias and limits of agreement split by tissue type and MI status are shown in table 1.

**Table 1.** Mean bias and limits of agreement for IDIF-AE compared to arterial sampling as a means of determining $V_\mathrm{T}$. Values in brackets show 95% CI.

Tissue	Cohort	Mean bias (ml cm$^ {-3}$)	Limits of agreement (ml cm$^ {-3}$)
Brain	Naive	$-$0.10 ($-$0.26, 0.06)	$-$0.57, 0.36 ($-$0.86, 0.65)
Brain	MI	$-$0.14 ($-$0.42, 0.14)	$-$0.96, 0.68 ($-$1.38, 1.11)
Heart	Naive	$-$0.79 ($-$2.34, 0.76)	$-$4.78, 3.19 ($-$7.29, 5.71)
Heart	MI	$-$1.67 ($-$4.06, 0.73)	$-$8.56, 5.23 ($-$12.12, 8.79)

**Figure 11.**
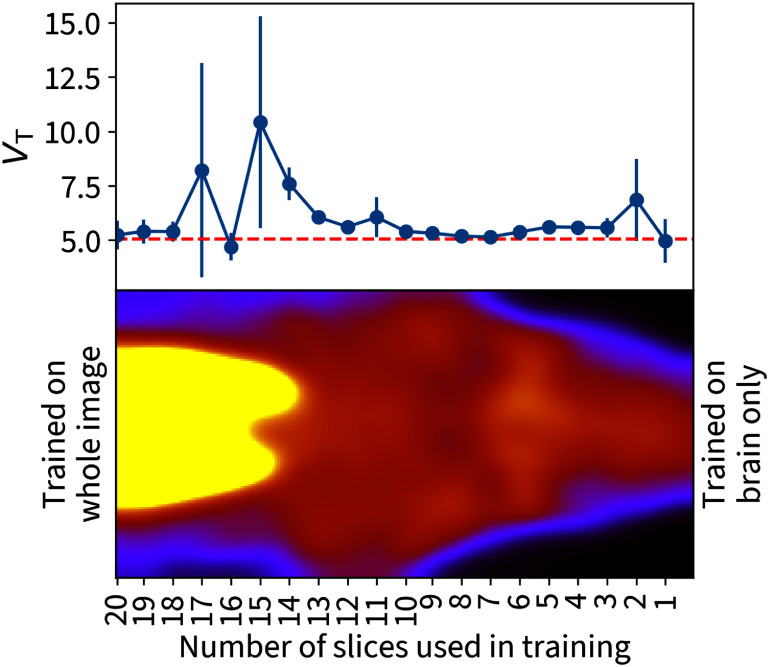
$V_\mathrm{T}$ values found using IDIF-AE with subsections of the image, compared to ground truth AIF $V_\mathrm{T}$ (dashed red line). The rightmost data point was found using a slice of the image containing only parts of the brain. Moving leftward, more slices were added until the leftmost data point was found using the entire image, including heart and lungs.

Figure 11 illustrates how much of the image is required to successfully train the IDIF-AE and extract a suitable input function. In the example shown, the IDIF-AE was able to recover an accurate $V_\mathrm{T}$ even when only a portion of the brain was included. This result was somewhat inconsistent; in many scans, at least a small section of the upper lungs was necessary to obtain a reasonable $V_\mathrm{T}$ estimate.

### [$^ {18}$F]SynVesT-1

3.2.

**Figure 12.**
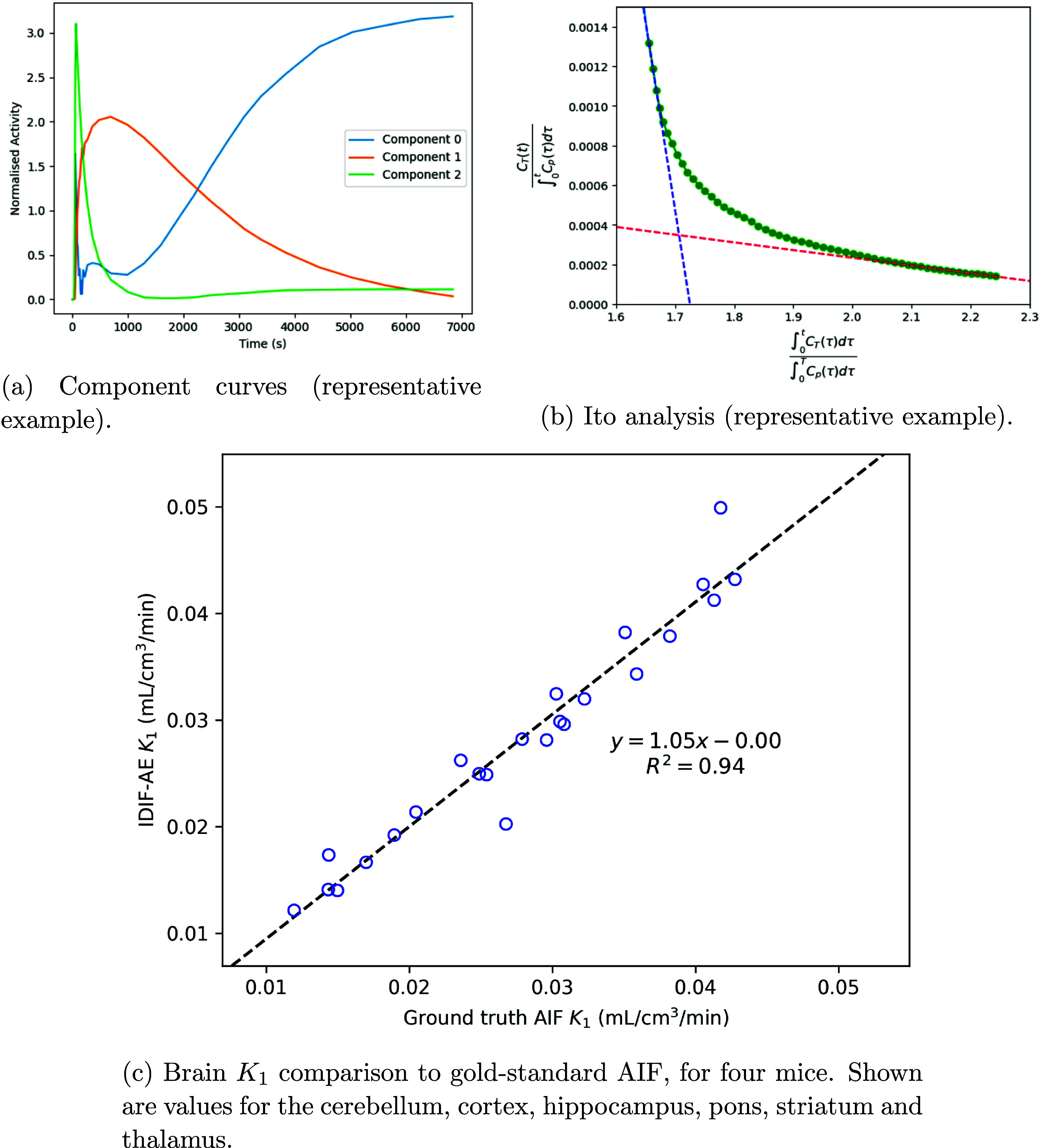
IDIF-AE performance tests for mouse scans with neurotracer [$^ {18}$F]SynVesT-1.

Figure 12(a) presents an example of the three component curves that the AE isolates for a mouse scan using the tracer [$^ {18}$F]SynVesT-1 tracer. The fastest responding curve (here component 0, black) is identified as being the IDIF-AE curve. Figures 12(b) and (c) show one aspect of the use of the IDIF-AE in downstream analysis. Here Ito analysis (Ito *et al* 2010) of the tissue curves (originating from the earlier study) is carried out using the IDIF-AE curve. The Ito linearisation for one image is shown in figure 12(b). This yields estimates for the microparameter $K_1$ and the macroparameter $V_\mathrm{T}$. The values of $K_1$ for various brain regions determined using our IDIF-AE curve are compared to those determined using the gold-standard AIF in figure 12(c).

The Pearson correlation coefficient between using gold-standard AIF with two-tissue kinetic modelling versus the IDIF-AE for $K_1$ was 0.95 and for $V_\mathrm{T}$ 0.75. The $V_\mathrm{T}$ values for IDIF-AE ranged between 1.3 and 2.6 and for AIF between 2.8 and 7.7. The mean bias in $K_1$ between the two methods was 0.0004 ml cm$^ {-3}$ min$^ {-1}$ (95% CI: $-$0.0005, 0.0013) with limits of agreement from $-$0.0026 to 0.0035 (95% CI: $-$0.0042 to 0.0051).

## 4. Discussion

While machine-learning based approaches have shown high accuracy in extracting IDIFs from pre-clinical dynamic PET scans, existing methods generally rely on identifying common features between a target scan and some set of training data (Kuttner *et al* 2020, 2021, 2024, Wang *et al* 2020, 2024, Jiang *et al* 2023, Whitehead *et al* 2023, Ferrante *et al* 2024, Moradi *et al* 2024, Chen *et al* 2025). This makes these methods difficult to generalise in clinical and pre-clinical research, where such training data is sparse and re-training for each new protocol is impractical. In this study, we addressed these limitations by developing a novel approach built on a custom undercomplete AE model (IDIF-AE) that is unique in that it uses only population-level assumptions about tracer kinetics and does not require a set of external training data. Using this approach, we successfully extracted input functions and kinetic parameters in [$^ {18}$F]LW223 rat and [$^ {18}$F]SynVesT-1 mouse scans. As the method cannot distinguish between metabolites, a population-based metabolite correction was applied to [$^ {18}$F]LW223 curves, while [$^ {18}$F]SynVesT-1 curves were left uncorrected to test limitations when suitable population-based corrections are not available.

As shown in figure 4, for [$^ {18}$F]LW223, the IDIF-AE input function reproduced the AUC around the peak better than a conventional manually-drawn region-of-interest IDIF taken from the left ventricle. This comparison was chosen as conventional IDIFs commonly underestimate the early peak, due to partial volume effects and low temporal resolution. The conventional IDIF underestimated the area-under-the-peak in 10 of 15 images, while only 7 images showed significant underestimation using the IDIF-AE. This indicates that the IDIF-AE may to some extent be capable of reducing the impact of partial volume effects.

This improved accuracy of the input function shape translated to more reliable kinetic modelling. As shown in figure 9, compared to a classical IDIF, the IDIF-AE $V_\mathrm{T}$ values mirrored the ground truth much more closely, with a relatively consistent offset pattern and a mean absolute percentage error of $\pm31.4\%$ (compared to $\pm53.9\%$ for the classical IDIF).

Notably, $V_\mathrm{T}$ values found using the IDIF-AE were more prone to underestimate rather than overestimate the ground truth. This indicates that the IDIF-AE often overstates the input function integral, which in turn may indicate that the IDIF-AE is also capturing part of the venous blood signal, changing the shape of the input function tail.

Bland-Altman plots comparing conventional IDIF, IDIF-AE and factor analysis as methods of determining $V_\mathrm{T}$ are shown in figure 10. The IDIF-AE produced markedly more consistent results than either of the other two methods. Of particular interest here is the superior performance compared to factor analysis which, while not commonly used with dynamic PET, is mathematically closely related to the IDIF-AE approach.

While continuous blood sampling is considered the most accurate method of determining the AIF, it may still introduce systematic and random measurement errors. For example, the input function measurement and PET acquisition must be manually synchronised. In addition, tracer-infused blood will take some time to permeate the vasculature, adding delay and dispersion, hence the measured AIF may not be timed together with the effective input function in large parts of the body. Because of this, a full match between image-derived and arterially-sampled input functions may not be achievable or required.

The machine learning model underpinning the IDIF-AE algorithm is newly initialised with randomised weights each time it is trained. Model training uses a gradient descent algorithm, which settles on a local minimum. Subsequent runs on identical data can therefore produce different results. To assess this phenomenon, we performed repeated IDIF-AE extractions on all scans in our dataset. We compared scans on the timing of the early peak, since here there is no need for normalisation, which would introduce additional errors. The results of this analysis are shown in figure 7, noting that the spread in early peak times was $\pm35$ s at most, or less than $\pm0.5\%$ of the total scan duration. For most images, the spread was far smaller, indicating good reproducibility.

Alongside reproducibility, the model was evaluated for its ability to generalise without overfitting. This is a key challenge in machine learning, where models can learn the training data too closely, including their noise patterns, and thus perform poorly with unseen data (Bourlard and Kabil 2022). While the IDIF-AE approach does not involve model invocation on unseen data, overfitting would lead the model to extract components from the random PET noise distribution rather than the underlying signal. As shown in figure 6, we found this to have a negligible effect.

The IDIF-AE model produces three primary curves in the image, of which one approximates the input function. The other curves average between tissue dynamics in different regions in the body and so do not have a clear physical interpretation. The model also assigns to each voxel contribution factors for each curve (compare equation (1)). As our data featured no voxels containing only blood, contribution factor and curve scaling was not possible without a blood sample for normalisation (as with conventional IDIFs). Figures 8(a) and (b) show the distribution of contribution factors in a representative scan.

We found that using the voxels with the highest input function contribution for scaling the input function (as opposed to using a blood sample) was unreliable, with very significant errors due to partial volume effects, small features, and noise in the image. However, treating these voxels as being fully filled with arterial blood is sufficiently accurate for a qualitative analysis of the distribution of contribution factors. Figure 8(a) shows that a large majority of voxels have no or almost no input function contribution. This is consistent with expectations, since the input function should only be present in voxels that contain parts of the vasculature. Another large group of voxels had contributions of around 1% of the maximum. These voxels were notably present in the brain. Again this is consistent with expectations, as the rat brain has a vascular volume of around 10 $\mu$l g$^ {-1}$ (Mandikian *et al* 2018) and specific gravity of around 1.0 (DiResta *et al* 1991), thus voxels in the brain should on average contain around 1% of the amount of blood in a fully-filled voxel.

Finally, we analysed the stability of our approach when limited data is available. Of particular interest was whether a usable IDIF-AE could be extracted from scans of the brain alone, where conventional IDIFs can generally not be used as even the largest available blood vessels (the carotid arteries) are too small not to suffer from severe partial volume effects. The results of this analysis for a representative scan are shown in figure 11. For some scans, such as the one shown, the IDIF-AE results are reasonable even when only including the brain, albeit with lower reproducibility (large differences between subsequent runs). For many scans, however, suitable performance was only achieved when at least a small section of the lungs was included. This still outperforms conventional IDIFs, as no suitable blood pools exist in that region, and leaves the possibility that performance is limited by the training algorithm and regulariser not selecting a sufficiently pure input function signal, rather than fundamental limits in the data imposed by resolution and partial volume effects.

Alongside thorough investigations of the TSPO tracer, we have also presented our attempts to estimate the IDIF-AE for four mice scanned with the tracer [$^ {18}$F]SynVesT-1. One of the key aspects which changes with this tracer is that it is metabolised by the mouse to a significant extent during the period of the scan. Ideally, the input functions and the tissue activity curves would both be corrected to reflect the breakdown of the tracer. To estimate the IDIF-AE for this tracer we found it necessary to adjust the regulariser settings. The IDIF-AE has a tendency to plateau at long times rather than to return to zero. Hence the extent to which the AE was steered towards monotonically decreasing weights was reduced.

In the current study, we have not made any attempt to correct the IDIF-AE for the decrease in the parent fraction of [$^ {18}$F]SynVesT-1 with time. Such an approach reflects the common situation that a detailed characterisation of the tracer breakdown is not available. We are well-able to determine the microparameter $K_1$ (figure 12(c)). The $V_\mathrm{T}$ from the IDIF-AE and Ito plot was strongly correlated with the $V_\mathrm{T}$ using the AIF and the 2TCM, albeit highly biased. This is in line with results seen in Xiong *et al* (2023) where group differences in $V_\mathrm{T}$ for mouse scans of [$^ {18}$F]SynVesT-1 were still present without correcting for radiometabolties in the IDIF.

This is unsurprising given that the determination of $K_1$ relies on the very early portion of the IDIF-AE (i.e. short times). At this stage in the scan hardly any metabolism of the tracer has taken place. By contrast, the determination of $V_\mathrm{T}$ predominantly relies on data from later times in the scan. Here the parent fraction of the tracer is low and hence our uncorrected IDIF-AE estimates carry heavy bias.

To summarise, we developed and tested a novel method for extracting IDIFs from dynamic PET scans. This method makes use of machine learning concepts and frameworks, but crucially does not require external training data. We adjusted this method to perform reliably on a dataset of rat PET scans with TSPO tracer [$^ {18}$F]LW223. We found that our novel IDIF-AE method outperformed conventional region-of-interest IDIFs in reproducing the ground truth AIF and deriving kinetic parameter $V_\mathrm{T}$. It appears that this method may be able to extract suitable input functions from brain-only scans in small animals. We also looked at the performance of the IDIF-AE with a second radiotracer, [$^ {18}$F]SynVesT-1. Here we showed the flexibility of this approach in how the regulariser can be adjusted for different kinetics. We found that for each new tracer, such adjustments may be necessary to account for the variation in kinetics.

Notable drawbacks of the IDIF-AE are the computational effort required (extraction can take several minutes on most machines for small animal scans) and the continued need for a single normalising arterial blood sample, in common with conventional IDIFs. While the method does identify averaged kinetics in tissue, it does not extract useful tissue curves for individual regions of interest for use in pharmacokinetic analysis. Finally, while the method has been shown to be adaptable, it has not yet been validated with other widely used radiotracers.

## 5. Conclusions

This study developed and validated a novel AE-based method for extracting the input function from dynamic PET scans. This method uses machine learning techniques, but does not require any external training data, making it particularly useful in a pre-clinical context where data is sparse and novel tracers are employed. The method was optimised and evaluated using a set of 15 [$^ {18}$F]LW223 rat scans with arterial blood sampling from a previous study. $V_\mathrm{T}$ values obtained via Logan plotting using the ground truth AIF, IDIF-AE and classical IDIF were compared, with the IDIF-AE method showing significantly closer agreement with the ground truth than the classical approach (mean absolute percentage error to AIF $\pm31.4\%$ for IDIF-AE and $\pm53.9\%$ for classical IDIF). Adaptability of the method for other tracers was also demonstrated using mouse scans with neuro PET tracer [$^ {18}$F]SynVesT-1. By providing a more accurate alternative to classical IDIFs, this method enables better quantification of pre-clinical PET studies without terminal continuous arterial sampling.

## Data Availability

These results are based on data acquired as part of earlier studies. The data cannot be made publicly available upon publication because they are owned by a third party and the terms of use prevent public distribution. The data that support the findings of this study are available upon reasonable request from the authors.
